# Characterization of *Frankia casuarinae* Mutants Defective in Vesicle Envelope Development

**DOI:** 10.1264/jsme2.ME25037

**Published:** 2025-09-27

**Authors:** Ken-ichi Kucho, Kosuke Taniyama

**Affiliations:** 1 Graduate School of Science and Engineering, Kagoshima University, 1–21–35 Korimoto, Kagoshima 890–0065, Japan; 2 Faculty of Science, Kagoshima University, 1–21–35 Korimoto, Kagoshima 890–0065, Japan

**Keywords:** nitrogenase, nitrogen fixation, oxygen, vesicle

## Abstract

*Frankia*, a nitrogen-fixing actinobacterium, forms a unique multicellular structure known as a vesicle that is dedicated to nitrogen fixation. The vesicle is surrounded by a thick hopanoid lipid envelope that acts as a barrier against oxygen penetration, preventing nitrogenase inactivation. Five mutants produced a similar number of vesicles to the wild type; however, they failed to fix N_2_. The thickness of vesicle envelopes was reduced in all five mutants, and the oxygen concentration increased inside the vesicles of four mutants. Therefore, these mutants were unable to fix N_2_ due to the inactivation of nitrogenase caused by oxygen penetration into the vesicles.

Nitrogen is a fundamental element that plays a crucial role in sustaining life for all living organisms. However, due to its stable triple bond, most organisms cannot utilize dinitrogen (N_2_) gas directly. Nevertheless, nitrogen-fixing (N_2_-fixing) bacteria possess the capability to convert N_2_ into ammonia (NH_3_) and incorporate it into organic compounds, such as amino acids. This process of N_2_ fixation allows for the incorporation of nitrogen into ecological systems and, thus, N_2_-fixing bacteria play a vital role in the global nitrogen cycle.

N_2_ fixation is catalyzed by the complex metalloenzyme nitrogenase, comprising dinitrogenase (NifDK) and dinitrogenase reductase (NifH) (Dixon and Kahn, 2004). Nitrogenase is highly susceptible to oxygen, potentially causing its inactivation. To address this issue, N_2_-fixing bacteria develop a range of strategies, including behavioral, physiological, and structural adaptations (Gallon, 1992).

*Frankia* spp. are N_2_-fixing multicellular actinobacteria. When exposed to NH_3_-depleted and aerobic conditions, *Frankia* differentiate a spherical multicellular structure known as a vesicle, which is dedicated to N_2_ fixation (Huss-Danell, 1997). These vesicles are surrounded by an envelope composed of multiple layers of hopanoid lipids (Berry *et al.*, 1993). The thick envelope functions as a protective barrier that prevents the penetration of oxygen. This barrier allows nitrogenase, which is expressed within the vesicles, to maintain its activity (Benson and Silvester, 1993). Genes related to vesicle development have not yet been identified, except for those associated with hopanoid lipid synthesis, which are ubiquitous in the microbial world (Kannenberg and Poralla, 1999).

By using 1-methyl-3-nitro-1-nitrosoguanidine (NTG) and gamma rays (GR) as mutagens, we isolated *Frankia casuarinae* mutants deficient in N_2_ fixation (Kucho *et al.*, 2017). The number of vesicles was markedly reduced in five mutants (<~10% of the wild type [WT]) and they were also markedly smaller (Asukai and Kucho, 2020). Therefore, these mutants were considered to have mutations in genes that trigger vesicle differentiation processes. In contrast, five other mutants (strains G17D5, G26C1, N3H4, N4H4, and N9D9) produced a similar number of vesicles to WT with a visibly normal morphology (Kucho *et al.*, 2017). However, they were unable to fix N_2_. In the present study, we characterized the vesicle phenotypes of these five mutants in more detail to gain insights into the functions of the genes responsible for the aberrant phenotypes.

We used *F. casuarinae* strain CcI3 as WT (Nouioui *et al.*, 2016). According to the procedure described in Asukai and Kucho (2020), we induced vesicle formation in WT and the five mutants (strains G17D5, G26C1, N3H4, N4H4, and N9D9), and exami­ned their vesicles under microscopes. Briefly, *Frankia* strains were propagated in NH_3_-repleted (N+) BAP-TN+ liquid medium (Kucho *et al.*, 2009) at 28°C, and cells were subsequently transferred to NH_3_-depleted (N-) BAP-TN-medium (Kucho *et al.*, 2009). Four days after being transferred to N-conditions, vesicles were observed with phase-contrast optical systems using the MT5310L microscope (Meiji Techno) for size measurements. Vesicle sizes were measured using the image ana­lysis software ImageJ (https://imagej.net/ij/index.html). To evaluate the envelope, vesicles were observed with dark-field optical systems using the MT5310L microscope (Meiji Techno) 2, 4, and 8 days after being transferred to N-conditions.

No significant differences were observed in vesicle sizes between the N4H4 mutant and WT (Fig. 1). The vesicle sizes of the G17D5, G26C1, N3H4, and N9D9 mutants were slightly smaller than those of WT, but were significantly larger (more than 1.7 times) than those of the N10E6 mutant, which showed a severe defect in vesicle size (Asukai and Kucho, 2020). Overall, the vesicle sizes of the five mutants were similar to those of WT.

When observed under a dark-field microscope, the thickness of a vesicle envelope is proportional to its brightness because the light effect is attributed to birefringence produced by structural layering of the envelope (Parsons *et al.*, 1987; Asukai and Kucho, 2020). We exami­ned the thickness of vesicle envelopes by classifying their brightness into three levels. Representative dark-field microscopy images of the vesicles at each level are shown in Fig. S1. Four days after being transferred to N-conditions, approximately half of the WT vesicles had a thick (level 3) envelope, while one-fourth had a moderate (level 2) envelope and the remaining one-fourth had a thin (level 1) envelope (Fig. 2A and S2). The frequency of the thick (level 3) envelope was significantly lower in all the mutants than in WT and was very low in G17D5 (4%), N4H4 (0%), and N9D9 (4%) (Fig. 2A and S2). All five mutants produced more vesicles with moderate (level 2) or thin (level 1) envelopes than WT. These results suggest the presence of mutations in genes involved in the development of vesicle envelopes in these mutants.

Since the five mutants may have exhibited a faster turnover rate or slower development rate of vesicles, we exami­ned vesicle envelopes at earlier and later time points. Two days after being transferred to N-conditions, most vesicles of the five mutants, as well as WT, had thin (level 1) envelopes (Fig. 2B), indicating that the turnover rate by these mutants was not faster than that of WT. Eight days after being transferred to N-conditions, 5% of WT vesicles had thick (level 3) envelopes (Fig. 2C). This frequency was markedly lower than that on day four (50%, Fig. 2A). Similar decreases in the frequency of level 3 vesicles were also observed in the G17D5, G26C1, N3H4, and N9D9 mutants (Fig. 2A and C). The N4H4 mutant did not produce any vesicles with a level 3 envelope at any time point. These results indicate that these mutants did not exhibit a slower rate of vesicle development.

The aberrant vesicle envelopes in these mutants were presumed to be unable to prevent the penetration of oxygen into the vesicles, leading to an increase in the concentration of oxygen inside the vesicles. To confirm this hypothesis, we stained the vesicles with the hypoxia-responsive fluorescence dye, MAR (Goryo Chemical). The fluorescence intensity of MAR increases as the oxygen concentration decreases. Vesicle development was induced according to the procedure described above, and they were then stained with 1‍ ‍μM MAR at room temperature in the dark for 10‍ ‍min. Fluorescence imaging was conducted using the ECLIPSE 90i microscope (Nikon) equipped with a filter set with excitation at 465–495‍ ‍nm, a dichroic mirror at 505‍ ‍nm, and emission collection at 515–555‍ ‍nm.

WT vesicles stained with MAR exhibited bright fluorescence (Fig. S3), indicating that the internal space of the vesicles was hypoxic. In contrast, the fluorescence of the mutant vesicles appeared to be lower than that of WT vesicles (Fig. S3). In a more precise evaluation, fluorescence intensities were quantified using the image ana­lysis software ImageJ. The G17D5, G26C1, N4H4, and N9D9 mutants exhibited significantly lower fluorescence intensity than WT (Fig. 3). The N9D9 mutant, which produced the highest number of vesicles with a thin (level 1) envelope (Fig. 2), exhibited the lowest fluorescence intensity (Fig. 3), suggesting the highest oxygen concentration within its vesicles. Therefore, these four mutants were unable to fix N_2_ because nitrogenase was inactivated by oxygen that penetrated into the vesicles.

In the N3H4 mutant, the thickness of the vesicle envelope was not as thin as in the other mutants (Fig. 2) and the concentration of oxygen inside the vesicles also appeared to be at a normal level (Fig. 3). In this mutant, a mutation in the glutamine-dependent NAD^+^ synthase gene (Francci3_3146 gene) was shown to disable its N_2_-fixing ability (Kucho *et al.*, 2023). This gene is involved in the synthesis of NAD(H), and a decrease in intracellular NAD(H) levels is considered to reduce respiration, leading to the loss of N_2_-fixing ability. A reduction in oxygen consumption by respiration may also contribute to the elevated concentration of oxygen inside vesicles.

These mutants may also carry mutations in genes related to the synthesis of hopanoid lipids, which are the main constituents of the vesicle envelope (Berry *et al.*, 1993). There is an operon in the *F. casuarinae* CcI3 genome at which hopanoid synthesis-related genes cluster (Francci3_1427 to Francci3_1434) (Alloisio *et al.*, 2010). The genomes of the N3H4, N4H4, and N9D9 strains had previously been resequenced, and no mutations were detected in the operon (Kucho *et al.*, 2017). In the present study, the genomes of the G17D5 and G26C1 strains were newly resequenced. Genomic DNA was purified using the CTAB method, as described by Kucho *et al.* (2009). Genome sequencing and variant detection were conducted by Gene Nex using the Illumina NovaSeq 6000 platform. However, no mutations were identified in the hopanoid synthesis operon in the two strains. Therefore, in these mutants, as yet unidentified genes involved in the development of vesicle envelopes or genes that regulate vesicle differentiation processes (such as transcription factors or components of signal transduction pathways) may be impaired.

Two laboratories previously reported the successful transformation of *Frankia* spp. (Gifford *et al.*, 2019; Pesce *et al.*, 2019). Using these methods, we will be able to identify the genes responsible for the mutant phenotypes through complementation experiments using a genomic library of WT *F. casuarinae*.

## Citation

Kucho, K., and Taniyama, K. (2025) Characterization of *Frankia casuarinae* Mutants Defective in Vesicle Envelope Development. *Microbes Environ ***40**: ME25037.

https://doi.org/10.1264/jsme2.ME25037

## Supplementary Material

Supplementary Material

## Figures and Tables

**Fig. 1.**
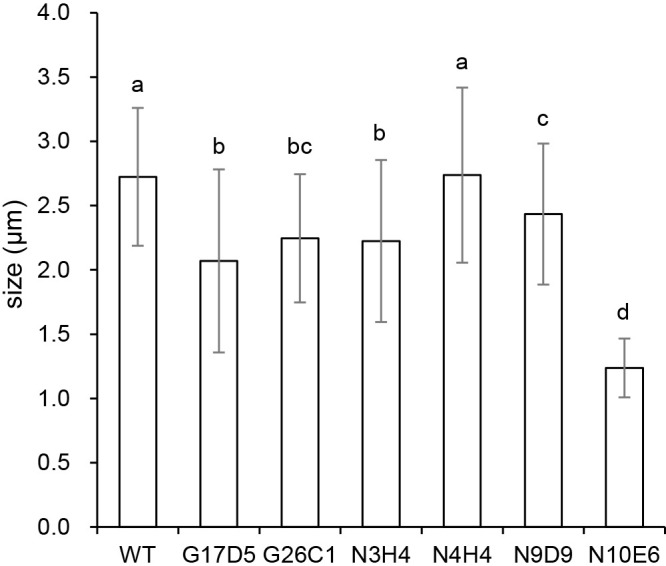
Size of vesicles. Average sizes were calculated using 60 to 240 vesicles obtained from two biological replicates. Bars represent standard deviations. Different letters indicate significant differences between strains (the Tukey-Kramer test, *P*<0.05).

**Fig. 2.**
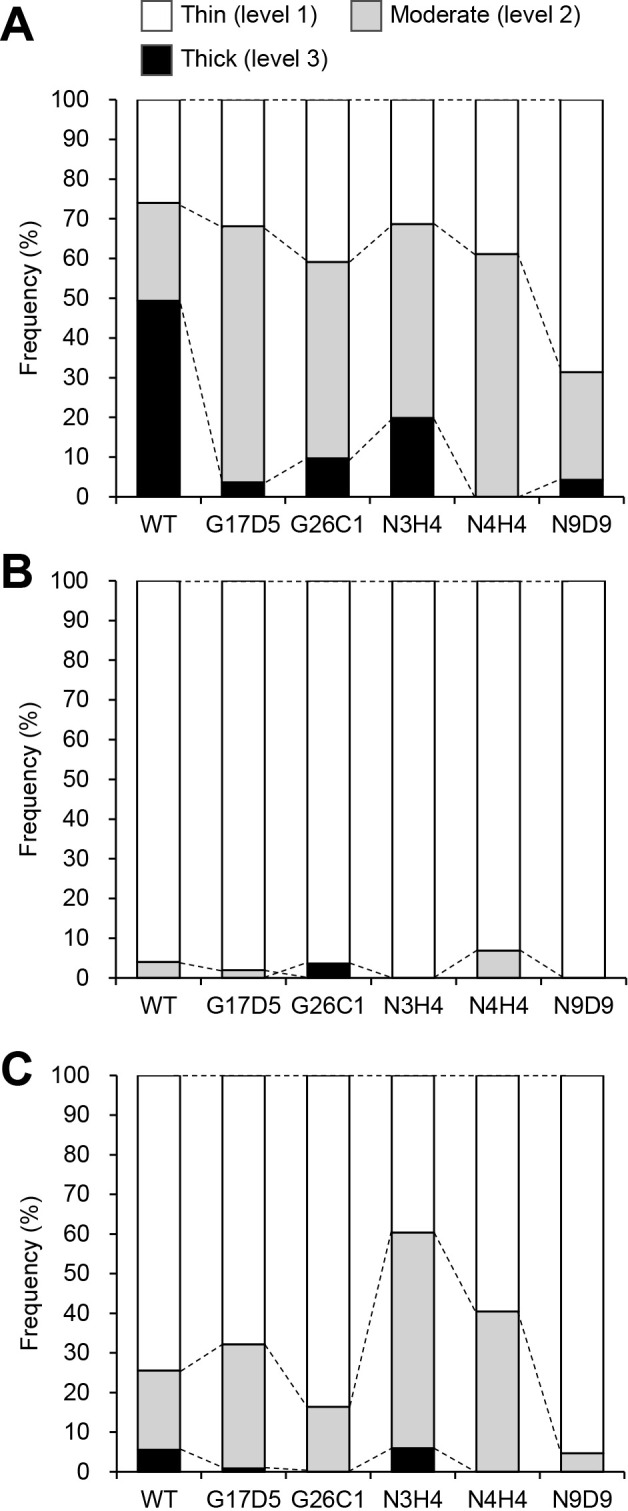
Thickness of vesicle envelopes 4 days (A), 2 days (B), and 8 days (C) after transferal to N-conditions. The frequencies of vesicles with thin (level 1, white box), moderate (level 2, gray box), and thick (level 3, black box) envelopes are shown. Frequencies were calculated using 50 to 193 vesicles obtained from two biological replicates.

**Fig. 3.**
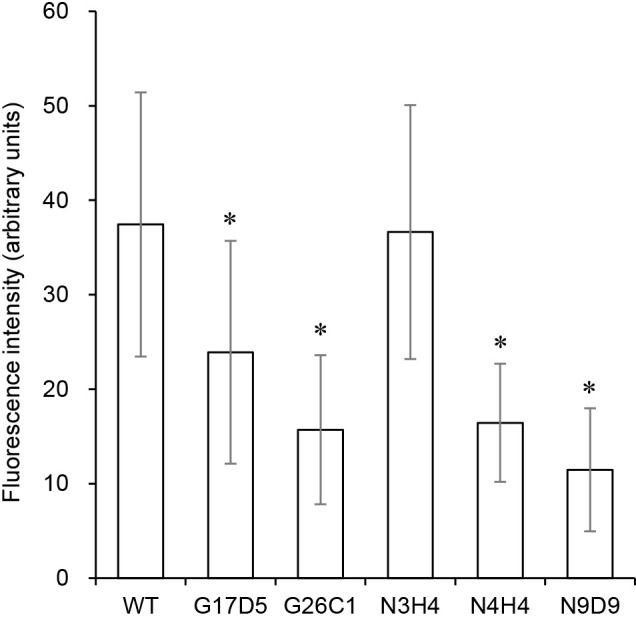
Fluorescence intensity of vesicles stained with MAR. Average intensities were calculated using 105 to 159 vesicles obtained from two biological replicates. Bars represent standard deviations. Asterisks indicate significant differences (*P*<0.05) from WT.
