# Supplementary material for: Characterization of Frankia casuarinae Mutants Defective in Vesicle Envelope Development
Source: Microbes Environ. 2025 Sep 27;40(3):ME25037. doi: 10.1264/jsme2.ME25037 (PMC12501873; doi:10.1264/jsme2.ME25037)
Supplement: Supplementary file 1 — Supplementary Material [file 40_25037_s1.pdf]

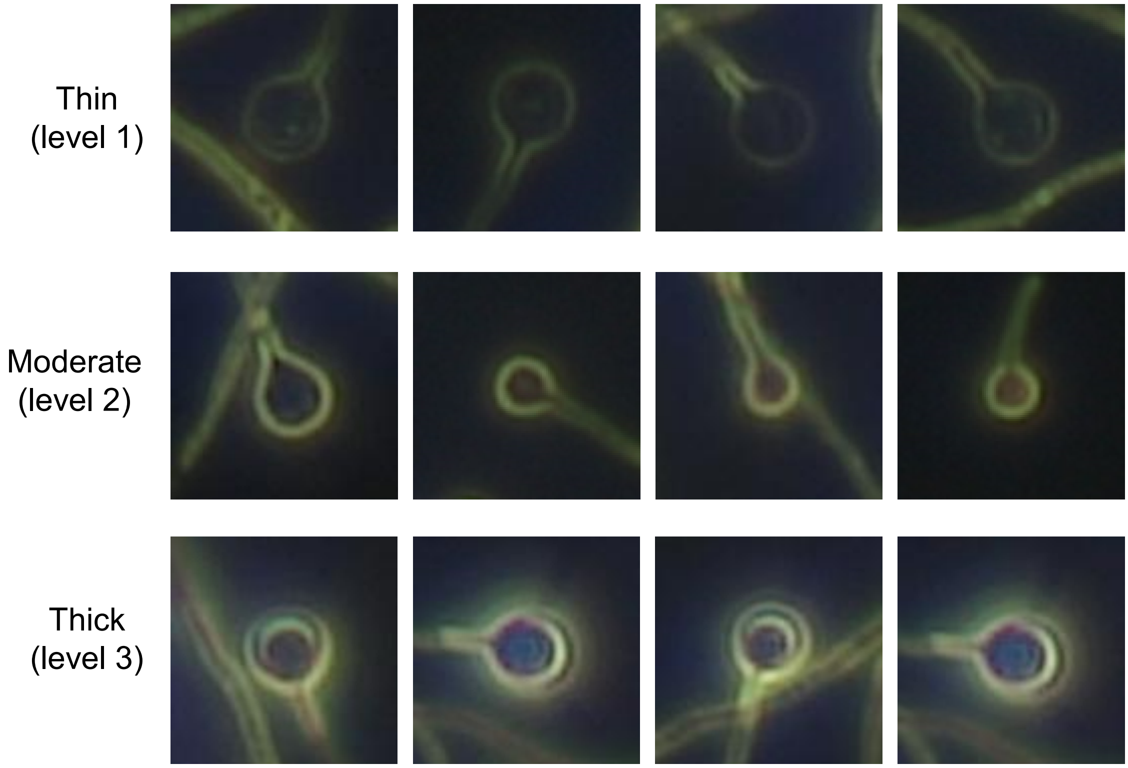

Fig. S1 Representative dark-field microscopic images of the vesicles with the different levels of envelope thickness.

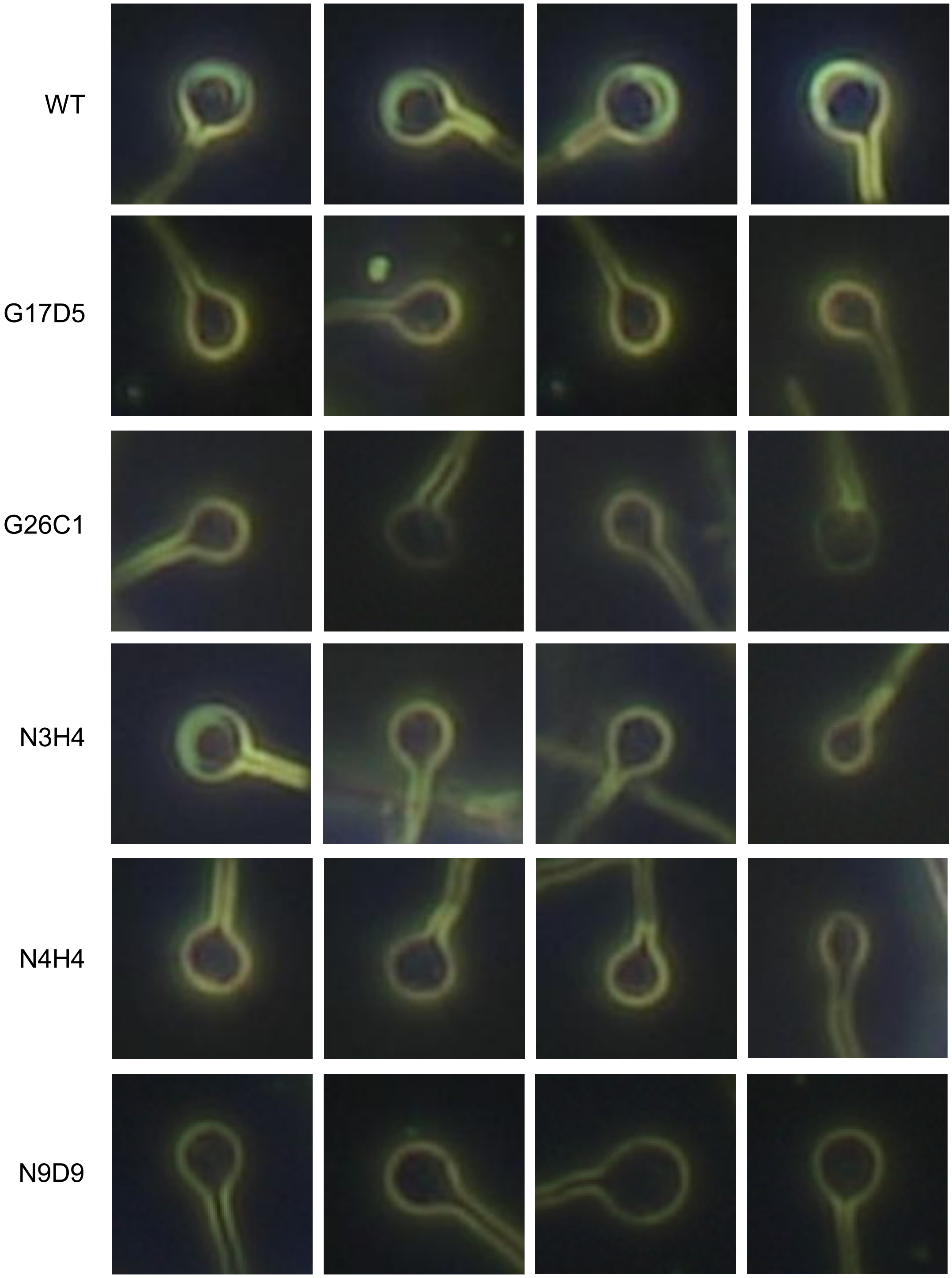

Fig. S2 Representative dark-field microscopic images of vesicles four days after transfer to the N- conditions.

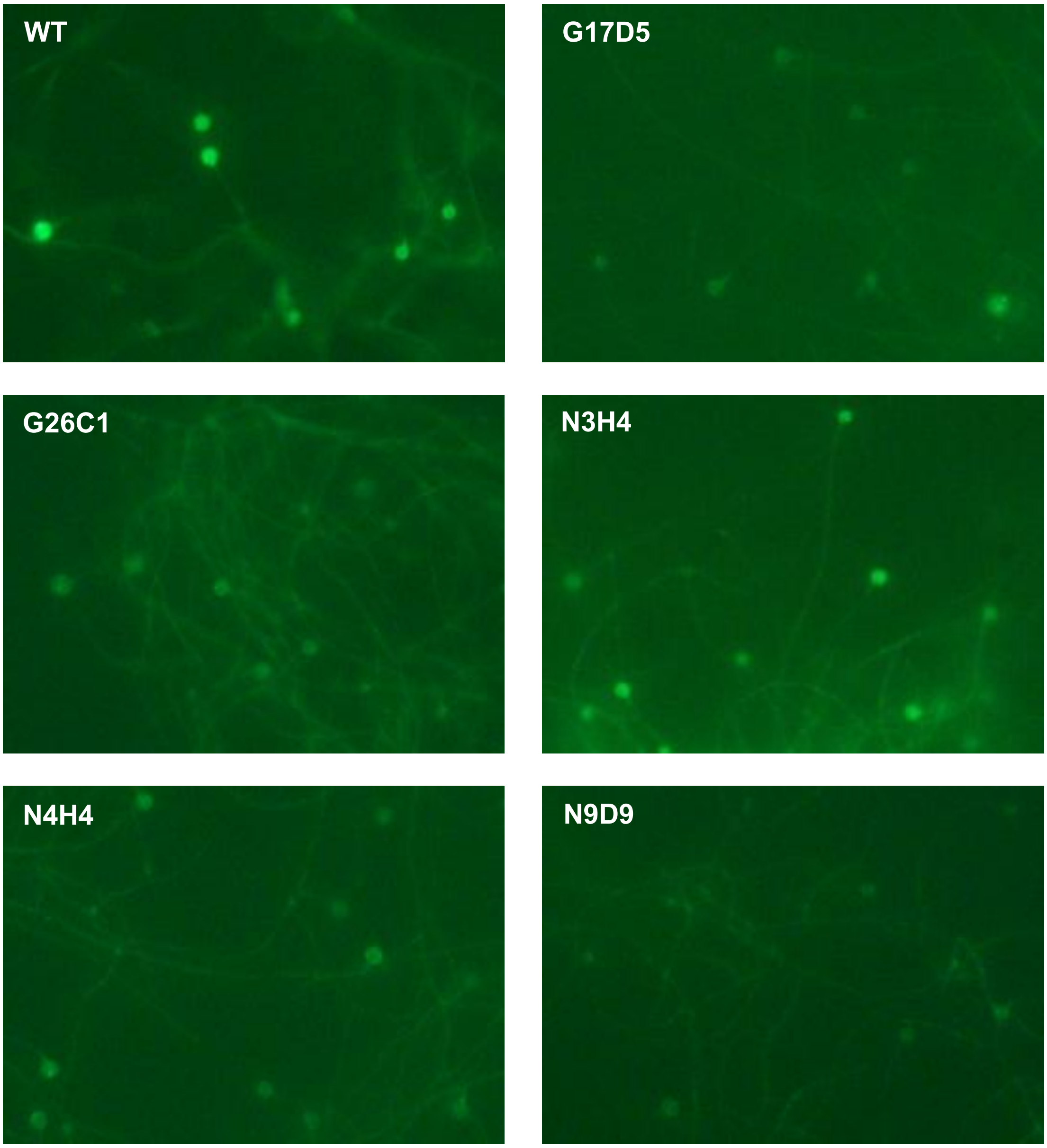

Fig. S3 Representative fluorescence microscopy images of vesicles stained with MAR.
